# O Primeiro Passo

**DOI:** 10.36660/abc.20220807

**Published:** 2022-11-23

**Affiliations:** 1 Universidade Federal do Rio de Janeiro Rio de Janeiro RJ Brasil Universidade Federal do Rio de Janeiro, Rio de Janeiro, RJ – Brasil

**Keywords:** Iniquidade de Gênero, Autoria, Publicações, Cardiologia

George Eliot e George Sand, romancistas do século XIX, eram pseudônimos de Mary Ann Evans e Amandine Aurore Lucile Dupin, respectivamente. Em comum, duas mulheres que para atingirem reconhecimento por seus textos, utilizavam nomes masculinos em suas obras, como bem relata Nodari em seu artigo publicado em 2021.^1^ Muito além do conhecimento histórico, analisar a disparidade de gênero na produção científica transcende a literatura e deve ser entendido como resultado de um processo de formação e desenvolvimento da sociedade. É inquestionável o modelo patriarcal, branco e com privilégios financeiros que, aliados a uma visão do “homem detentor da razão e a mulher detentora da emoção”,^2^ ajudaram a construir o modelo de ciência que, de maneira equivocada, afastou as mulheres do campo científico.

Pensar este modelo como algo pertencente ao passado não é adequado. Dados da Pesquisa Nacional por Amostra de Domicílios (PNAD), do Instituto Brasileiro de Geografia e Estatística (IBGE) de 2019, expõe uma realidade social que apresenta reflexos na formação da cientista.^3^ As mulheres dispensam duas vezes mais tempo aos afazeres domésticos por semana em relação aos homens da mesma faixa etária (21,4 versus 11 horas), têm menor remuneração que seus pares do sexo masculino (77% da renda masculina), e naquelas com idade entre 25 e 49 anos que convivem no mesmo domicílio que uma criança de até três anos, o percentual de inserção no mercado de trabalho é menor que o dos homens na mesma situação (59,6% versus 89,2%).^3^

Apesar do número crescente de mulheres na medicina e da formação universitária generalista mais jovem, a cardiologia ainda é uma especialidade com predominância masculina.^4,5^ Em 2020, a relação homem/mulher era 2,21, ao contrário de outras especialidades como dermatologia, pediatria e endocrinologia, com relações inferiores a 1.^4^ Este padrão observado no Brasil também é encontrado nos Estado Unidos^6^ e na Europa,^7^ com baixa representatividade global feminina na cardiologia ou em posições de liderança dentro da especialidade.

Após a especialização, o caminho segue em direção à pós-graduação para a formação docente com a consequente e natural liderança em áreas de pesquisa. Novamente, o cenário não sofre alterações e as mulheres representam 46% do corpo docente superior.^3^ Este dado é, na prática, refletido no estudo de Oliveira-Ciabati et al., que demonstrou que mais de 60% da composição docente da Universidade de São Paulo é do sexo masculino, o que também contribui para o aumento da disparidade de gênero nas publicações.^2^

Neste contexto, o estudo publicado nos Arquivos Brasileiros de Cardiologia é fundamental para o entendimento do meio acadêmico brasileiro.^8^ Nele, os autores abordam de maneira bastante elucidativa a disparidade de gênero nas publicações das revistas Arquivos Brasileiros de Cardiologia (ABC Cardiol) e International Journal of Cardiovascular Sciences (IJCS), de 2000 a 2019.

Entre os 1555 artigos publicados nas revistas que representam as maiores fontes de estudos na cardiologia do país, os autores observaram que as mulheres apresentaram predominância apenas na primeira autoria e apenas na revista IJCS (53%), enquanto homens predominaram na primeira autoria nos ABC Cardiol (58%) e como autores seniores em ambas (75% no ABC Cardiol e 59% no IJCS).^8^ De maneira geral, sabe-se que a primeira autoria reflete o principal responsável pelo artigo em questão, enquanto o último autor, ou sênior, reflete o líder da linha de pesquisa responsável pelo trabalho mais abrangente, capaz de gerar outras publicações.

Outra observação feita pelos autores refere-se à evolução temporal da predominância de gênero nos campos autorais. Apesar de estarem sub-representadas nas revistas, entre 2000 e 2019, no ABC Cardiol, houve aumento na primeira autoria feminina dos artigos, de 12% para 44%. Enquanto última autoria, e, portanto, autoras seniores, o aumento foi mais modesto, de 16% em 2000 para 27% em 2019, na mesma revista.^8^

Entre os fatores ressaltados pelos autores que podem contribuir para esta disparidade de gênero, estão a visão estereotipada de que apenas o homem representa o cientista de sucesso, e a maternidade.^5,8^ Embora o primeiro possa ser inconsciente e o segundo inerente ao gênero, ambos devem ser considerados na discussão sobre disparidade nas publicações cientificas e nenhum dos dois, isoladamente é consistente com os resultados observados.

O padrão notado no estudo^8^ não é exclusivo das publicações nacionais,^7^ e pode ser observado também em ensaios clínicos randomizados na cardiologia indexados no *PubMed* entre 2011 e 2020,^9^ e em diretrizes do Colégio Americano (American College of Cardiology/American Heart Association – ACC/AHA), da Sociedade de Canadense (Canadian Cardiovascular Society – CCS), e da Sociedade Europeia de Cardiologia (European Society of Cardiology – ESC), entre 2006 e 2020.^10^ Em ambos os estudos que avaliaram as publicações mencionadas, a sub-representatividade das mulheres se mantém, seja na autoria principal, seja como líder de pesquisa.

Por fim, a desigualdade de gênero encontrada nas publicações científicas tem origem multifatorial e inter-relações complexas, refletindo provavelmente o comportamento da sociedade. Iniquidades existentes anteriores à formação no ensino superior, desigualdades de renda e oportunidades devido à estereótipos inconsistentes, maternidade, dupla jornada de trabalho e sexismo estrutural, são apenas alguns entraves no sinuoso caminho da mulher até a autoria ou liderança científica. Para mudar este cenário, é preciso reconhecer o problema, e desta forma, a contribuição do estudo^8^ é essencial. O caminho em direção a equidade é longo, mas o primeiro passo foi dado.^8^
