# Supplementary material for: O Primeiro Passo
Source: Arq Bras Cardiol. 2022 Nov 23;119(6):968–9. [Article in Portuguese] doi: 10.36660/abc.20220807 (PMC9814807; doi:10.36660/abc.20220807)
Supplement: Translation

# Translation: The First Step

**Affiliations:** 1 Rio de Janeiro RJ Brazil Universidade Federal do Rio de Janeiro, Rio de Janeiro, RJ – Brazil

**Keywords:** Gender Inequality, Authorship, Publications, Cardiology

George Eliot and George Sand, 19th-century novelists, were pen names for Mary Ann Evans and Amandine Aurore Lucile Dupin, respectively. In common, two women who, to achieve recognition for their texts, used male names in their works, as portrayed by Nodari in his article published in 2021.^1^ Far beyond historical knowledge, analyzing gender disparity in scientific production transcends literature and must be understood as the result of a process of formation and development of society. The patriarchal, white model with financial privileges is unquestionable, which, combined with a vision of the “man with reason and the woman with emotion”,^2^ helped to build the model of science that, in a wrong way, kept women away from the scientific field.

To think of this model as something belonging to the past is inadequate. Data from the National Household Sample Survey (PNAD) from the Brazilian Institute of Geography and Statistics (IBGE) in 2019 exposes a social reality that reflects on the formation of the scientist.^3^ Women spend twice as much time on housework per week compared to men of the same age group (21.4 versus 11 hours), earn less than their male peers (77% of male income), and in those aged between 25 and 49 years old who live in the same household with a child up to three years old, the percentage of insertion in the labor market is lower than that of men in the same situation (59.6% versus 89.2%).^3^

Despite the growing number of women in medicine and the younger generalist university education, cardiology is still a specialty with a male predominance.^4,5^ In 2020, the male/female ratio was 2.21, unlike other specialties such as dermatology, pediatrics and endocrinology, with ratios lower than 1.^4^ This pattern observed in Brazil is also found in the United States^6^ and Europe,^7^ with low global female representation in cardiology or leadership positions within the specialty.

After the specialization, the path goes towards the postgraduate course for teacher training with the consequent and natural leadership in research areas. Again, the scenario does not change; women represent 46% of the superior faculty.^3^ This data is, in practice, reflected in the study by Oliveira-Ciabati et al., which showed that more than 60% of the professors at the University of São Paulo are male, which also contributes to the increase in gender disparity in schools publications.^2^

In this context, the study published by *Arquivos Brasileiros de Cardiologia* is fundamental for understanding the Brazilian academic environment.^8^ In it, the authors approach in a very elucidative way the gender disparity in the publications of *Arquivos Brasileiros de Cardiologia* (ABC Cardiol) and the International Journal of Cardiovascular Sciences (IJCS) from 2000 to 2019.

Among the 1555 articles published in journals that represent the largest sources of studies in cardiology in the country, the authors observed that women predominated only in the first authorship and only in the IJCS journal (53%), while men predominated in the first authorship in the ABC Cardiol (58%) and as senior authors in both (75% in ABC Cardiol and 59% in IJCS).^8^ Generally, it is known that the first author reflects the main responsible for the article in question, while the last author, or senior, reflects the leader of the research line responsible for the most comprehensive work, capable of generating other publications.

Another observation made by the authors refers to the temporal evolution of gender predominance in authorship fields. Despite being underrepresented in magazines, between 2000 and 2019, in ABC, there was an increase in the first female authorship of articles, from 12% to 44%. As the last author, and therefore senior authors, the increase was more modest, from 16% in 2000 to 27% in 2019, in the same jornal.^8^

Among the factors highlighted by the authors that may contribute to this gender disparity are the stereotyped view that only men represent the successful scientist and motherhood.^5,8^ Although the first may be unconscious and the second inherent to the genre, both must be considered in the discussion about the disparity in scientific publications and neither, in isolation, is consistent with the observed results.

The pattern noted in the study^8^ is not unique to national publications.^7^ It can also be observed in randomized clinical trials in cardiology indexed in PubMed between 2011 and 2020^9^ and in the American College of Cardiology/American Heart guidelines Association – ACC/AHA), the Canadian Cardiovascular Society (CCS), and the European Society of Cardiology (ESC), between 2006 and 2020.^10^ In both studies that evaluated the publications mentioned, the underrepresentation of women remains in the main authorship or as a research leader.

Finally, the gender inequality found in scientific publications has a multifactorial origin and complex interrelationships, probably reflecting the behavior of society. Existing inequities prior to higher education training, inequalities in income and opportunities due to inconsistent stereotypes, motherhood, double shifts and structural sexism are just a few obstacles on women’s winding path to scientific authorship or leadership. To change this scenario, it is necessary to recognize the problem, and in this way, the contribution of the study^8^ is essential. The path toward equity is long, but the first step has been taken.^8^
